# Supplementary material for: Dupilumab Leads to Clinical Improvements including the Acquisition of Tolerance to Causative Foods in Non-Eosinophilic Esophagitis Eosinophilic Gastrointestinal Disorders
Source: Biomolecules. 2023 Jan 5;13(1):112. doi: 10.3390/biom13010112 (PMC9856177; doi:10.3390/biom13010112)
Supplement: Supplementary file 1 [file biomolecules-13-00112-s001.zip › biomolecules-1967500-supplementary.pdf]

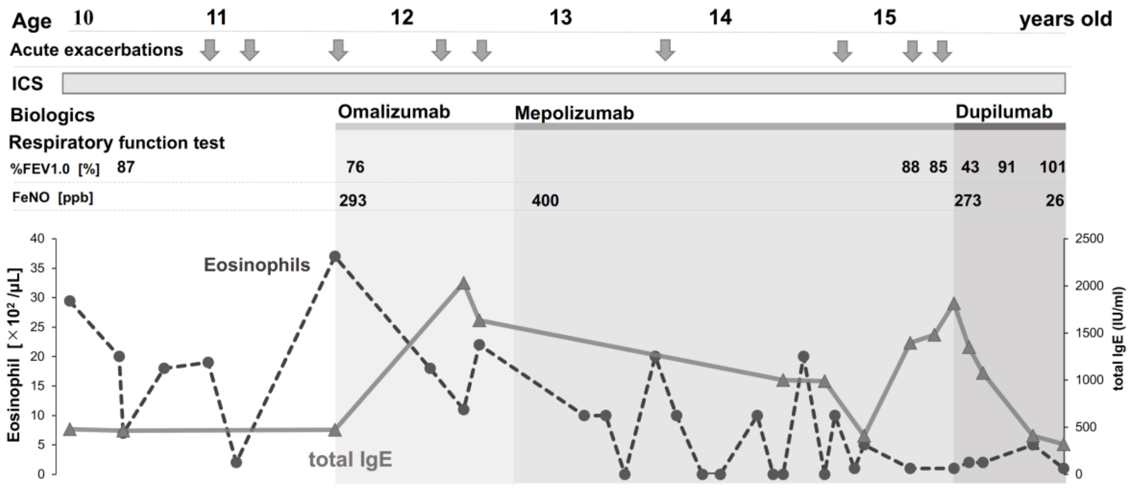

Supplementary Figure S1. Clinical course of asthma during biologics therapy. Peripheral blood eosinophil counts (gray broken line) and total IgE levels (solid line) are graphically illustrated. ICS, inhaled corticosteroid; FEV, forced expiratory volume; FeNO, fractional exhaled nitric oxide.
